# Breastfeeding trajectories for preterm infants over the first 6 months of life in England 2010–2020: surveys using large representative birth samples

**DOI:** 10.1136/bmjpo-2024-002912

**Published:** 2024-10-21

**Authors:** Ilana Levene, Sian Harrison, Fiona Alderdice, Maria A Quigley

**Affiliations:** 1 National Perinatal Epidemiology Unit, Nuffield Department of Population Health, University of Oxford, Oxford, UK

**Keywords:** Breastfeeding, Infant, Neonatology, Epidemiology

## Abstract

**Background:**

Breastmilk is the optimal source of nutrition for infants, particularly preterm infants. Preterm infants face unique feeding challenges and these change with the birth gestation of the infant. Preterm infants’ feeding outcomes may have been affected differently than term infants by the SARS-CoV-2 pandemic. The objective of this study was to describe the feeding trajectories of preterm infants in the first 6 months of life compared with term infants and compare these across prepandemic and pandemic periods.

**Methods:**

Data were extracted from the 2010 UK Infant Feeding Survey and the English National Maternity Surveys of 2018 and 2020. Original survey weights were used. Infants were divided by gestation into term (37+0 to 42+6 weeks’ postmenstrual age), late preterm (34+0 to 36+6 weeks’ postmenstrual age) and a lower gestation group (23+0 to 33+6 weeks’ postmenstrual age). Modified Poisson regression, log-rank tests and survival curves were used to analyse feeding outcomes by gestational age.

**Results:**

Late preterm infants had lower adjusted relative risk (aRR) of exclusive breastmilk at 6 weeks of age compared with term infants in 2010 and 2020 but not in 2018. In 2010, aRR was 0.47 (95% CI 0.29 to 0.74), in 2018 aRR was 0.86 (95% CI 0.67 to 1.10) and in 2020 aRR was 0.57 (95% CI 0.41 to 0.81). There was no evidence of differences in feeding outcomes between infants born less than 34 weeks’ postmenstrual age and term infants.

**Conclusion:**

Late preterm infants had worse breastfeeding outcomes than term infants in 2010, but the inequity was reduced or eliminated in 2018. In 2020, during the early SARS-CoV-2 pandemic, the inequity had re-emerged. Late preterm infants appear to be a particularly vulnerable population in relation to breast feeding. In future emergencies and natural disasters, late preterm infants should receive additional focus and resources to support breastfeeding establishment.

**WHAT IS ALREADY KNOWN ON THIS TOPIC**

- Late preterm infants often have poorer breastfeeding outcomes than term infants. There are little data comparing long-term breastfeeding outcomes between gestational age groups.

**WHAT THIS STUDY ADDS**

- Between 2010 and 2018, inequities in breastfeeding outcomes between late preterm and term infants significantly improved. However, they recurred, and in some outcomes worsened, in 2020. Infants born less than 34 weeks’ postmenstrual age had feeding outcomes that were more similar to term infants.

**HOW THIS STUDY MIGHT AFFECT RESEARCH, PRACTICE OR POLICY**

- Late preterm infants appear to be a vulnerable population in relation to breast feeding. In future emergencies and natural disasters, late preterm infants should receive additional focus and resources to support breastfeeding establishment.

## Introduction

 Breastmilk is the optimal nutrition for infants, particularly preterm infants.^1^ Feeding outcomes for preterm infants are commonly reported at discharge from hospital,^2^,^4^ but there is less evidence available for the months following, or how this compares to term infants. Preterm infants face feeding challenges and these change with birth gestation. Those born under 34 weeks postmenstrual age (PMA) are unlikely to fully feed orally from birth so the expression of milk is important, which mothers often find difficult.^5^,^7^ These infants are generally cared for in neonatal units, which increases the separation between mother and infant but also brings more potential for staff support. Motivation can be higher when infants are very premature—for example, 51% of pregnant women planning to formula feed reported that they would give their babies breastmilk if born early or sick.^8^

By contrast, late preterm infants (born at 34–36 weeks’ PMA) often have medical needs such as jaundice and hypoglycaemia that can interfere with breast feeding even when they remain with their mothers.^9 10^ Late preterm infants are less likely than term infants to initiate breast feeding within an hour of birth^11^ and more likely to be readmitted to hospital with feeding difficulties.^9^ Late preterm babies have reduced stamina and oromotor immaturity compared with term infants but may show similar behaviour^9 12^; their need for feeding support may, therefore, be underestimated.^13^

Several large national surveys, the Infant Feeding Survey 2010, and the National Maternity Surveys 2018 and 2020, are sources of information about infant feeding in the UK.^14^,^16^ The subset of preterm infants naturally nested within them has not been fully explored. In particular, the 2020 National Maternity Survey, commissioned to explore the effect of the SARS-CoV-2 pandemic on perinatal experiences,^16^ presents the opportunity to assess how the pandemic influenced preterm feeding trajectories.

The objective of this study is to describe the feeding trajectories of preterm infants in the first 6 months of life compared with term infants and compare these across prepandemic and pandemic periods.

## Methods

### Survey design

Data were extracted from three surveys. Similarities and differences in survey design are shown in table 1. Most notably the 2010 dataset was derived from three survey waves, the latest at 9 months after birth, whereas the 2018 and 2020 datasets relate to single surveys at 6 months after birth. Full methodology and reports are available for each survey.^14^,^16^

**Table 1:** Survey design comparisons

	2010 Infant Feeding Survey	2018 National Maternity Survey	2020 National Maternity Survey
Population of births	August to October 2010	October 2017	May 2020
Countries included	England, Scotland, Wales, Northern Ireland	England	England
Sample identified	Random sample, oversampling those from the lowest index of multiple deprivation quintile	Random sample	Random sample
Sample source	Office for National Statistics	Office for National Statistics	Office for National Statistics
Number of participants approached	30 760	15 528	15 972
Number of surveys	Three	One	One
Type of survey	Paper and electronic options	Paper and electronic options	Paper and electronic options
Survey infant target ages	6 weeks, 4–6 months, 8–10 months	6 months	6 months
Focus	Infant feeding	Perinatal experiences	Perinatal experiences
Multiple birth approach	Full answers for the first child only	Full answers for the first child only	Full answers for the first child only
Variables used for weighting	Maternal age, index of multiple deprivation, stage 1 survey response characteristics	Maternal age, marital status, country of birth, index of multiple deprivation, region of England, parity	Maternal age, marital status, country of birth, index of multiple deprivation, region of England, parity

#### Definitions

All surveys asked about breast feeding similarly, including reference to direct breast feeding and giving expressed breastmilk by any route. Surveys used identical wording to ask about the initiation of complementary feeds. In this report, exclusive breastmilk feeding is defined as an infant who has received only breastmilk and never infant formula, complementary feeds or any other liquid by the specified time point.

Term infants were born at 37+0 weeks’ PMA or above. Late preterm infants were born between 34+0 and 36+6 weeks’ PMA. The lowest category of preterm infants includes those born between 23+0 and 33+6 weeks’ PMA. The latter category spans three subcategories usually referred to as moderately preterm (32+0 to 33+6 weeks’), very preterm (28+0 to 31+6 weeks’) and extremely preterm (less than 28+0 weeks’). Infants born at less than 23+0 weeks or more than 42+6 weeks’ PMA were excluded.

#### Statistical analysis

Weighted descriptive statistics are reported for baseline characteristics and infant feeding prevalence at selected time points. Survey weights were provided within each original dataset and are described elsewhere.^14^,^16^ From the 2010 Infant Feeding Survey, only data from England are included, for comparability with National Maternity Surveys.

Modified Poisson regression estimated relative risks (RR) for the association of birth gestation and infant feeding outcomes within each survey. Important sociodemographic and obstetric influences on infant feeding were identified from the literature and included in the multivariable regression models regardless of their significance (maternal age, education, ethnic background, index of multiple deprivation, parity, multiple birth, smoking status and caesarean birth).

Weighted survival curves were constructed using the reported time to last breastmilk or last exclusive breastmilk. Participants were censored at 24 or 26 weeks of actual age (exclusive and any breastmilk, respectively). Log-rank test and relative hazards are presented to compare gestational age groups within individual surveys, and separately to compare surveys within gestational age groups. Survival curves and log-rank tests were adjusted for the five variables showing the greatest association with preterm birth, from those identified in the literature (maternal education, index of multiple deprivation, parity, multiple birth and caesarean). Stata SE V.18.0 was used for analysis.

#### Patient and public involvement

Patient and public involvement is described in the original survey reports.^14^,^16^

## Results

### Participant flow chart and characteristics

**Figure 1:**
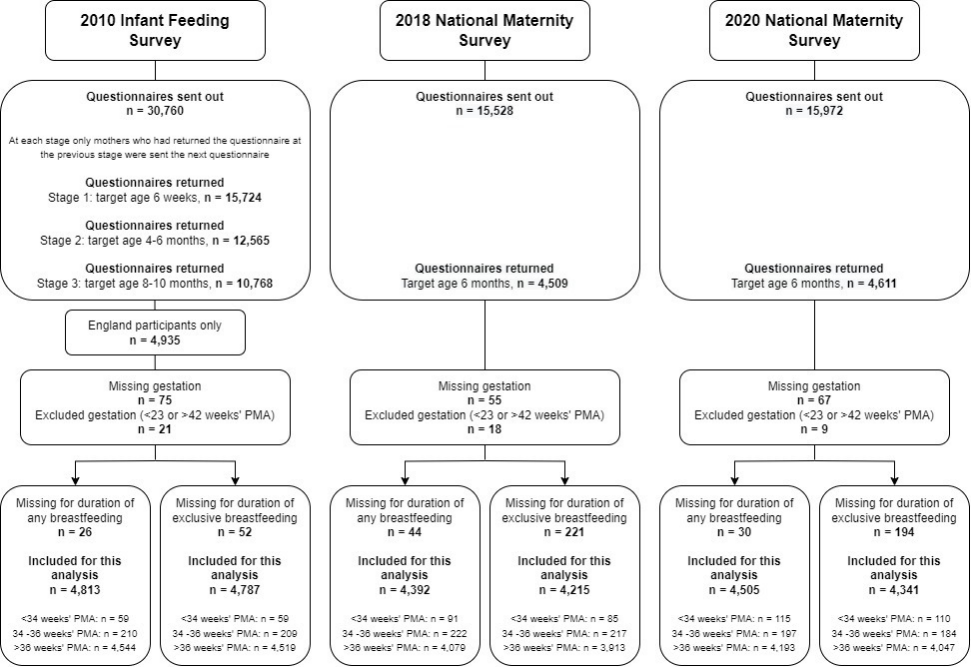
Flow chart of participants. PMA, postmenstrual age.

Figure 1 shows participant data flow. Final response rate was 35% in 2010 and 29% in 2018 and 2020.

Table 2 shows baseline characteristics. Over consecutive time periods, participants were on average older (29.0% were 30–35 years old in 2010, 34.0% in 2018 and 34.9% in 2020); less likely to leave education aged under 17 years (17.8% in 2010, 14.5% in 2018 and 13.9% in 2020) and less likely to be of White British ethnicity (77.7% in 2010, 70.3% in 2018 and 68.1% in 2020). They were less likely to have smoked during pregnancy (20.1% in 2010, 8.5% in 2018 and 6.4% in 2020) and more likely to have had caesarean birth (22.5% in 2010, 26.7% in 2018 and 29.9% in 2020; all trends p<0.001).

**Table 2:** Baseline demographic and perinatal characteristics

	Infant Feeding Survey 2010(n=4813)*	National Maternity Survey 2018(n=4425)*	National Maternity Survey 2020(n=4523)*
Maternal age at birth; n (%)‡			
<20 years	250 (5.2)	93 (2.1)	74 (1.7)
20 to <25 years	890 (18.6)	519 (11.8)	496 (11.1)
25 to <30 years	1325 (27.6)	1092 (24.8)	1073 (23.9)
30 to <35 years	1388 (29.0)	1496 (34.0)	1565 (34.9)
35 to <40 years	760 (15.9)	932 (21.2)	989 (22.1)
≥40 years	182 (3.8)	272 (6.2)	289 (6.4)
Ethnic background; n (%)‡			
White British	3602 (77.7)	2984 (70.3)	3031 (68.1)
White other	363 (7.8)	487 (11.5)	601 (13.5)
Black	192 (4.2)	162 (3.8)	233 (5.2)
Asian	356 (7.7)	371 (8.7)	390 (8.8)
Other	49 (1.1)	127 (3.0)	73 (1.6)
Mixed ethnicity	77 (1.7)	113 (2.7)	121 (2.7)
Age mother left full time education; n (%)‡			
<17 years	849 (17.8)	634 (14.5)	619 (13.9)
17–18 years	1441 (30.2)	1146 (26.2)	1260 (28.2)
≥19 years	2477 (52.0)	2591 (59.3)	2584 (57.9)
Index of Multiple Deprivation quintile; n (%)			
1 (most deprived)	1356 (28.2)	1177 (26.6)	1147 (25.4)
2	1079 (22.4)	987 (22.3)	982 (21.7)
3	874 (18.2)	816 (18.5)	878 (19.4)
4	769 (16.0)	759 (17.2)	811 (17.9)
5 (least deprived)	729 (15.2)	686 (15.5)	705 (15.6)
Primiparous; n (%)‡	2542 (52.8)	1949 (45.0)	2048 (46.2)
Multiple birth; n (%)*§*	61 (1.3)	97 (2.2)	67 (1.5)
Caesarean birth; n (%)‡	1085 (22.5)	1176 (26.7)	1342 (29.9)
Ever smoked; n (%)‡	1945 (41.4)	1520 (34.5)	1453 (32.6)
Smoked during pregnancy; n (%)‡	947 (20.1)	371 (8.5)	284 (6.4)
Gestation at birth in weeks’ PMA; mean (SD)‡	39.5 (1.9)	39.1 (2.2)	39.0 (2.2)
Gestation at birth category*§*			
<34 weeks’ PMA; n (%)	59 (1.2)	92 (2.1)	121 (2.7)
34–36 weeks’ PMA (‘late preterm’); n (%)	236 (4.9)	246 (5.6)	221 (4.9)
≥37 weeks’ PMA (‘term’); n (%)	4531 (93.9)	4086 (92.4)	4180 (92.4)
Median gestation at birth (IQR) for those <34 weeks’ PMA	31 (30–33)	31 (28–32)	31 (28–32)
Median gestation at birth (IQR) for those 34–36 weeks’ PMA	36 (35–36)	36 (35–36)	36 (35–36)
Mother stayed in hospital for ≤48 hours after birth†; n (%)			
Birth<34 weeks’ PMA	24 (42.6)	46 (51.8)	58 (52.5)
Birth 34–36 weeks’ PMA (‘late preterm’)	65 (29.2)	81 (32.9)	69 (32.3)
Birth ≥37 weeks’ PMA (‘term’)‡	3019 (69.8)	2957 (75.2)	3144 (79.7)

* This is the number of participants with data available for initiation of breast feeding and demographic information. Some demographic/perinatal variables have a higher level of missing data than others. Results are weighted using each survey’s individual weightings.

† This applies only to participants giving birth in hospital or a midwife-led unit or birth unit separate from hospital.

‡ p<0.001 between years.

§ p<0.05 between years.

PMApostmenstrual age

A slightly higher proportion of respondents had infants born at less than 34 weeks’ gestation in later surveys (1.2% in 2010, 2.1% 2018 and 2.7% 2020; p<0.05). The proportion of respondents with late preterm infants was similar across the years (4.9%, 5.6% and 4.9%, respectively). The minimum gestation for those born under 34 weeks’ gestation was 26 weeks’ in 2010, 24 weeks’ in 2018 and 23 weeks’ in 2020, while the median remained 31 weeks’ in all periods (table 2).

## Gestational age at birth and infant feeding outcomes

Table 3 shows the weighted prevalence of breast feeding at birth, 6 weeks and 6 months of age for gestational age groups across the three surveys. The unadjusted and adjusted RR (aRR) of each feeding outcome for each category of preterm infants is compared with term infants. The prevalence estimates of any and exclusive breastmilk were higher at each consecutive time point for term infants only. For both categories of preterm infants, the prevalence estimates were higher in 2018 than in 2010 but lower in 2020 than 2018 (with the exception of any breastmilk at 6 months for those born under 34 weeks’ gestation infants, which was highest in 2020).

**Table 3:** Prematurity and breastmilk feeding; prevalence and RR

	2010			2018			2020		
	Prevalence	RR, 95% CI	aRR, 95% CI	Prevalence	RR, 95% CI	aRR, 95% CI	Prevalence	RR, 95% CI	aRR, 95% CI
Breastfeeding initiation									
≥37 weeks	82%	1	1	85%	1	1	86%	1	1
34–36 weeks	76%	0.93(0.84 to 1.02)	0.97(0.88 to 1.07)	82%	0.96(0.89 to 1.04)	0.97(0.90 to 1.04)	77%	0.90(0.82 to 1.00)†	0.89(0.81 to 0.98)†
<34 weeks	87%	1.06(0.95 to 1.18)	1.10(0.97 to 1.24)	92%	1.07(1.01 to 1.14)†	1.09(1.00 to 1.19)	80%	0.94(0.82 to 1.07)	0.98(0.87 to 1.09)
Exclusive breastmilk at 6 weeks of age									
≥37 weeks	27%	1	1	41%	1	1	40%	1	1
34–36 weeks	12%	0.43(0.28 to 0.67)*	0.47(0.29 to 0.74)*	31%	0.75(0.59 to 0.96)*	0.86(0.67 to 1.10)	19%	0.48(0.34 to 0.68)*	0.57(0.41 to 0.81)*
<34 weeks	20%	0.76(0.46 to 1.26)	0.93(0.54 to 1.58)	42%	1.02(0.77 to 1.35)	1.31(0.99 to 1.72)	32%	0.80(0.59 to 1.08)	1.02(0.77 to 1.34)
Any breastmilk at 6 weeks of age									
≥37 weeks	58%	1	1	64%	1	1	66%	1	1
34–36 weeks	45%	0.78(0.66 to 0.92)†	0.84(0.71 to 0.98)†	61%	0.95(0.84 to 1.08)	0.98(0.88 to 1.10)	54%	0.81(0.69 to 0.96)†	0.82(0.70 to 0.97)†
<34 weeks	61%	1.05(0.84 to 1.31)	1.13(0.87 to 1.46)	66%	1.03(0.85 to 1.24)	1.10(0.92 to 1.31)	56%	0.85(0.70 to 1.04)	0.95(0.82 to 1.11)
Exclusive breastmilk at 6 months of age									
≥37 weeks	4%	1	1	17%	1	1	19%	1	1
34–36 weeks	2%	0.43(0.17 to 1.06)	0.60(0.26 to 1.39)	12%	0.70(0.47 to 1.05)	0.90(0.61 to 1.33)	9%	0.49(0.28 to 0.85)†	0.51(0.27 to 0.95)†
<34 weeks	3%	0.69(0.17 to 2.88)	0.39(0.06 to 2.58)	11%	0.68(0.33 to 1.38)	0.95(0.43 to 2.09)	10%	0.55(0.33 to 0.91)*	0.78(0.48 to 1.26)
Any breastmilk at 6 months of age									
≥37 weeks	37%	1	1	46%	1	1	50%	1	1
34–36 weeks	26%	0.69(0.54 to 0.89)†	0.74(0.57 to 0.94)†	37%	0.80(0.66 to 0.99)†	0.87(0.71 to 1.05)	25%	0.50(0.37 to 0.66)*	0.53(0.41 to 0.70)*
<34 weeks	28%	0.76(0.50 to 1.15)	0.80(0.53 to 1.22)	33%	0.71(0.51 to 0.98)†	0.74(0.54 to 1.03)	38%	0.76(0.59 to 0.99)†	0.96(0.79 to 1.18)

Prevalence is weighted with individual survey weights*.*

Adjusted for maternal education, age, ethnic background, multiple birth, parity, birth mode and smoking status in pregnancy. ≥37 weeks is referred to as ‘term’ in the text and 34–36 weeks as ‘late preterm’*.*

* p<0.001.

† p<0.05.

aRRadjusted relative riskRRrelative risk

After adjustment for demographic and perinatal factors, in 2010 late preterm infants were less likely than term infants to be breastfed (aRR 0.84, 95% CI 0.71 to 0.98, p<0.05) or exclusively breastfed (aRR 0.47, 95% CI 0.29 to 0.74, p<0.001) at 6 weeks of age, and less likely to be breastfed at 6 months of age (aRR 0.74, 95% CI 0.57 to 0.94, p<0.05). In 2020, these relationships were similar at 6 weeks of age and greater at 6 months of age (for any breastmilk aRR=0.53, 95% CI 0.41 to 0.70, p<0.001). However, in 2018, there was no difference in aRR for feeding outcome for late preterm infants compared with term infants at any of the studied time points (table 3, p>0.05).

No significant differences were seen in adjusted analysis comparing feeding outcomes for those born under 34 weeks’ gestation and term infants (table 3, p>0.05).

## Survival curves

**Figure 2:**
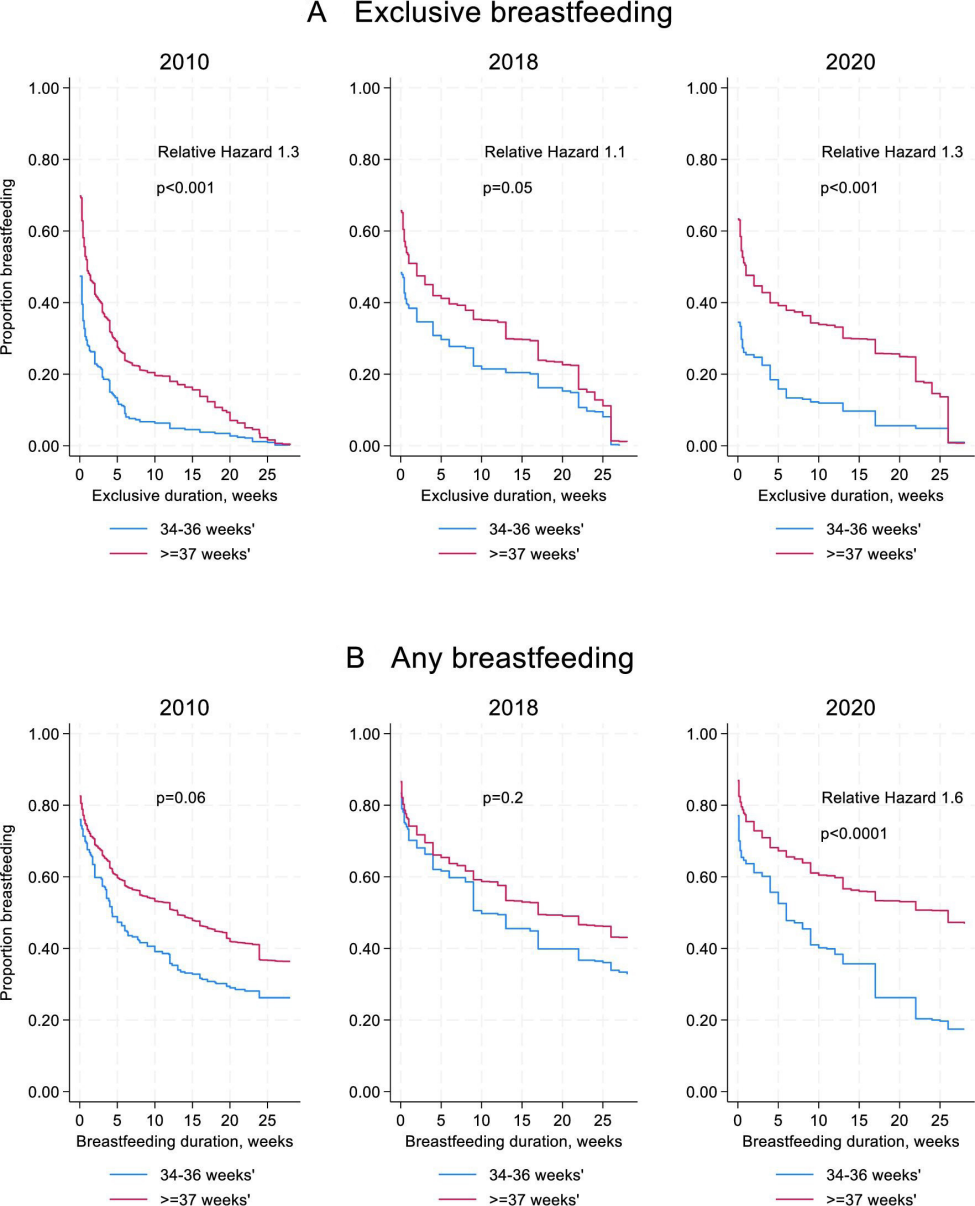
Adjusted survival curves for exclusive breast feeding (**A**) and any breast feeding (**B**), comparing term and late preterm infants. Results are weighted using each survey’s individual weightings and adjusted for maternal education, Index of Multiple Deprivation, caesarean birth, parity and multiple birth.

The adjusted survival curves in figure 2 show the breastfeeding trajectory in the first 6 months after birth in each time period. Late preterm infants had a higher relative hazard for cessation of exclusive breast feeding in all time periods, compared with term infants, although this was at the borderline of statistical significance in 2018. Late preterm infants had a higher relative hazard for cessation of any breast feeding in 2020 only.

**Figure 3:**
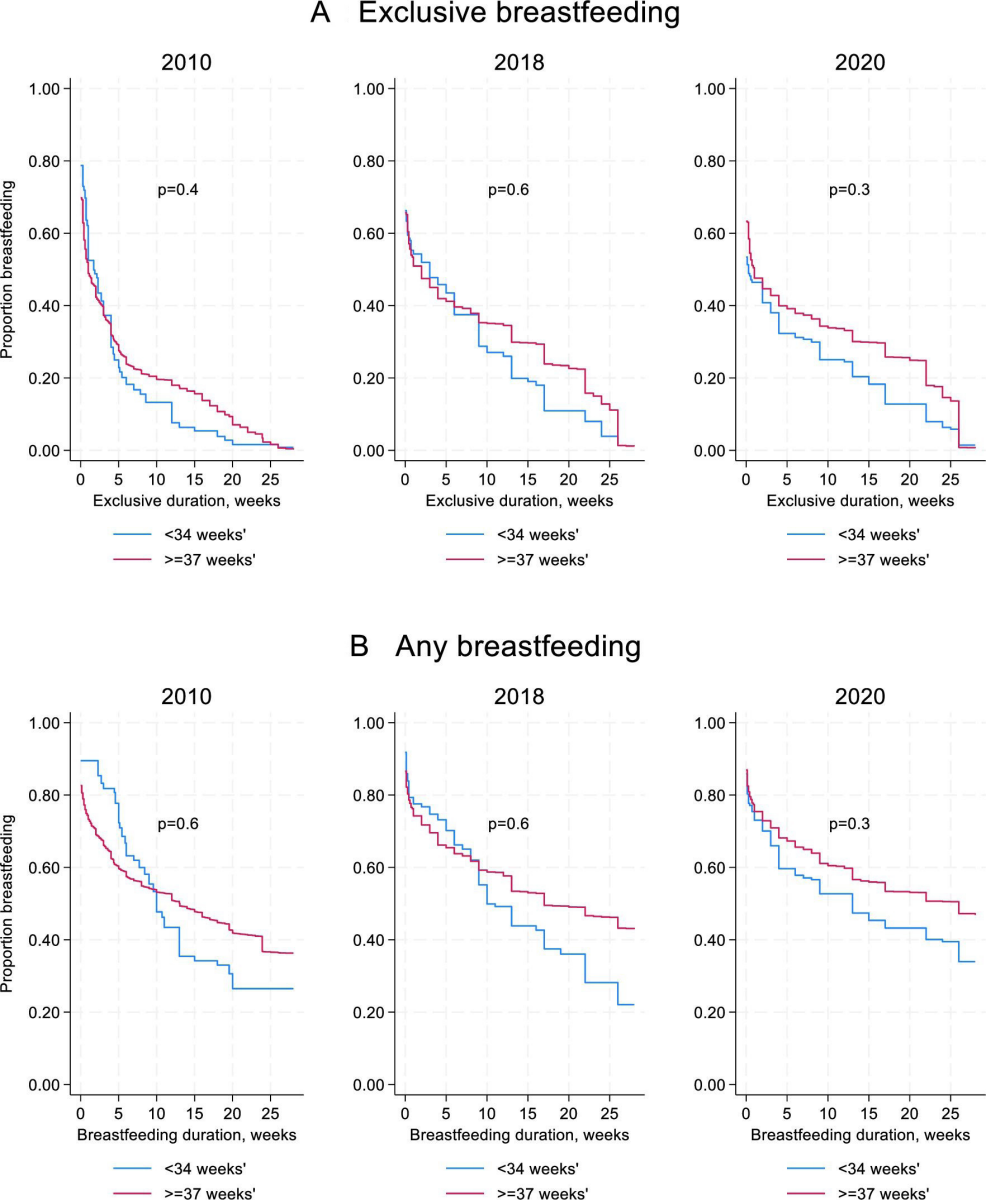
Adjusted survival curves for exclusive breast feeding (**A**) and any breast feeding (**B**), comparing term infants and those born under 34 weeks’ gestation. Results are weighted using each questionnaire’s individual weightings and adjusted for maternal education, Index of Multiple Deprivation, caesarean birth, parity and multiple birth.

Survival analysis did not show any differences in relative hazard for cessation of any or exclusive breast feeding for infants born under 34 weeks’ gestation in any time period, compared with term infants (figure 3).

## Comparing time periods

Median duration of breast feeding and exclusive breast feeding in each survey period is shown in table 4, for all infants and separately for those who initiated breast feeding. Only term infants saw consistent improvement in outcomes between consecutive time periods (for those who initiated breast feeding, duration increased from median 24 weeks in 2010 to 25 weeks in 2018 and 26 weeks in 2020, p<0.001).

**Table 4:** Breastfeeding and exclusive breastfeeding duration for all infants and for infants who had initiated breastmilk feeding

	2010	2018	2020
All infants			
Last breastmilk feed in weeks; median (IQR)			
<34 weeks’ PMA	10 (4–28)	10 (1–26)	9 (0–26)
34–36 weeks’ PMA	4 (0–28)	9 (0–26)	6 (0–22)
≥37 weeks’ PMA	13 (1–28)*	17 (1–26)*	22 (1–28)
Last exclusive breastmilk feed in weeks; median (IQR)			
<34 weeks’ PMA	1 (0–4)	3 (0–13)	0 (0–9)
34–36 weeks’ PMA	0 (0–2)†	0 (0–9)†	0 (0–3)
≥37 weeks’ PMA	1 (0–6)*	2 (0–18)	1 (0–22)
Infants who initiated breast feeding			
Last breastmilk feed in weeks; median (IQR)			
<34 weeks’ PMA	11 (5–28)	13 (5–26)	22 (4–26)
34–36 weeks’ PMA	12 (3–28)	17 (5–27)	13 (4–26)
≥37 weeks’ PMA	24 (4–28)*	25 (6–27)*	26 (6–28)
Last exclusive breastmilk feed in weeks; median (IQR)			
<34 weeks’ PMA	2 (1 – 5)	4 (0–13)	2 (0–15)
34–36 weeks’ PMA	0 (0–4)†	1 (0–17)†	0 (0–6)
≥37 weeks’ PMA	3 (0–12)*	4 (0–22)	4 (0–22)

* p<0.001.

† p<0.05 (compared with the subsequent time period, within each gestational age group).

PMApostmenstrual age

Late preterm infants increased exclusive breastfeeding duration between 2010 and 2018, but this decreased between 2018 and 2020 (for those who initiated breast feeding, from median 0 weeks in 2010 to 1 week in 2018, p=0.02, then to 0 weeks in 2020, p=0.03). Online supplemental figure 1 shows the feeding trajectories of gestation subgroups by survey period.

### Discussion

This analysis shows that late preterm infants had worse breastfeeding outcomes (with a variety of measures) than term infants in 2010, but this inequity was reduced or eliminated in 2018. However, in 2020, during the early SARS-CoV-2 pandemic, the inequity was the most evident of the three time points, with lower breast feeding and exclusive breast feeding for late preterm infants at all ages examined and in survival curve analysis.

The main strength of this analysis is the use of very large representative birth samples, reducing selection bias. Biases due to differences between responders and non-responders were at least partially corrected for using survey weights. Previous publications have shown that non-responders for the three surveys were younger and more disadvantaged.^14^,^16^ In 2018 and 2020, weighted results were validated against routinely collected national data, for example, showing the expected prevalence of caesarean and prematurity. Other variables such as ethnic background were less well matched despite weighting.^15 16^

As expected due to social trends, mothers’ age, education and prevalence of caesarean birth rose over time, while smoking and white British background declined. This is a continuation of established trends in UK society.^17^ The direction of these changes (apart from the rise in caesarean birth) would be expected to correlate with improved breastfeeding outcomes.^17^ In addition, variables such as maternal age and ethnic background are associated with both preterm birth^18^ and breastfeeding outcomes.^19^ Therefore, the statistical adjustment for sociodemographic and perinatal variables is a key strength for the validity of the conclusions.

The main limitation of this analysis is the small size of the preterm subgroups within each survey, particularly those born under 34 weeks’ gestation. This limits statistical power to identify any true differences in feeding trajectories for these infants. In addition, there were some differences in survey design between 2010 and 2018/2020, due to the different underlying aims of an infant feeding survey compared with maternity surveys, and higher response rate in 2010. This should introduce a higher level of caution in interpreting changes between 2010 and 2018. However, the key finding of worsening breastfeeding outcomes, and increased inequity, for late preterm infants between 2018 and 2020 remains a reliable conclusion as the methods of these two surveys were extremely similar.

The proportion of subgroups of preterm infants in the sample is similar to Office for National Statistics data; 5% of births in 2010 were late preterm, 5.7% in 2018 and 5.3% in 2020; 2.2% of births in 2010 were at less than 34 weeks’ gestation, 2.2% in 2018 and 2.1% in 2020.^20 21^ The breastfeeding prevalence figures reported here are higher than those reported in governmental routinely collected statistics, but the trends are consistent across time and routine statistics may underestimate breast feeding.^16^

This report is consistent with multiple global cohorts showing that late preterm infants have worse breastfeeding outcomes than term infants.^9 22 23^ However, the narrowing and elimination of inequity of feeding outcomes by gestation in 2018 is encouraging and requires further attention. This type of improvement has not to our knowledge been reported in other contexts or countries.

It is possible that the improvement was due to increased breastfeeding support^13^ as the number of UNICEF Baby Friendly Initiative (BFI) accredited hospitals continues to rise, as well as the move towards transitional care models for late preterm infants.^24^ Not receiving enough help with feeding in hospital has a more detrimental effect on breastfeeding duration in late preterm infants than term infants.^18^

The recurrence of inequity during the early period of the SARS-CoV-2 pandemic is revealing. The 2020 National Maternity Survey showed that women were less likely to attend antenatal classes, received fewer postnatal home visits and had less health professional breastfeeding support in this period.^19^ More women were discharged within 24 and 48 hours of birth,^16^ although our data suggest that this predominantly impacted mothers of term infants. There was a 9% increase in emergency department attendance for feeding-related problems in infants during the first year of the pandemic period in England, supporting the existence of a gap in the usual system of feeding support.^25^

Whereas term mother–infant pairs were resilient to these challenges, maintaining or improving their breastfeeding outcomes,^19^ this report shows that late preterm mother–infant pairs were not. These findings suggest that late preterm infants are a particularly vulnerable population requiring specific focus when there are extra environmental challenges. Interestingly, when prematurity is used as a generic category, combining late preterm infants with those of lower gestation, these important relationships may be obscured.^19^

The number of infants born under 34 weeks’ gestation in these survey populations was small, meaning that there was little statistical power to detect any differences in their breastfeeding outcomes. However, their breastfeeding prevalence estimates are useful because there are few studies focused on long-term breast feeding in this population, despite their higher morbidity and the known potential of breastmilk to modify morbidity.^26 27^ The data presented here suggest that these lower gestation infants do not share the same set of vulnerabilities as late preterm infants, but there are potential trends for poorer feeding outcomes to manifest after the first 3 months of life, in comparison to term infants. This would be consistent with the higher motivation to provide breastmilk^8^ and increased contact with health professionals mentioned previously when infants are in the neonatal unit. Such a hypothesis cannot be proved or disproved within the current sample size.

In summary, this report suggests that significant progress in improving inequities of breastfeeding outcomes was made for late preterm infants between 2010 and 2018, but this was to a large extent reversed in 2020. Therefore, only term infants have seen a consistent improvement or maintenance of breastfeeding outcomes between 2010 and 2020. Late preterm infants appear to be a vulnerable population in relation to breast feeding but it is encouraging that the inequality in their outcomes can be reduced. In future emergencies and natural disasters, late preterm infants should receive additional focus and resources to support breastfeeding establishment. Further analysis of the reasons for the improvements seen in 2018 and examination of whether outcomes have since returned to the 2018 baseline are needed.

## supplementary material

10.1136/bmjpo-2024-002912 online supplemental file 1

## Data Availability

Data may be obtained from a third party and are not publicly available.
