# Supplementary material for: Breastfeeding trajectories for preterm infants over the first 6 months of life in England 2010–2020: surveys using large representative birth samples
Source: BMJ Paediatr Open. 2024 Oct 21;8(1):e002912. doi: 10.1136/bmjpo-2024-002912 (PMC11499770; doi:10.1136/bmjpo-2024-002912)
Supplement: online supplemental file 1 [file bmjpo-8-1-s001.pdf]

Supplementary Figure 1: Adjusted breastfeeding survival curves over time, within each gestational age group

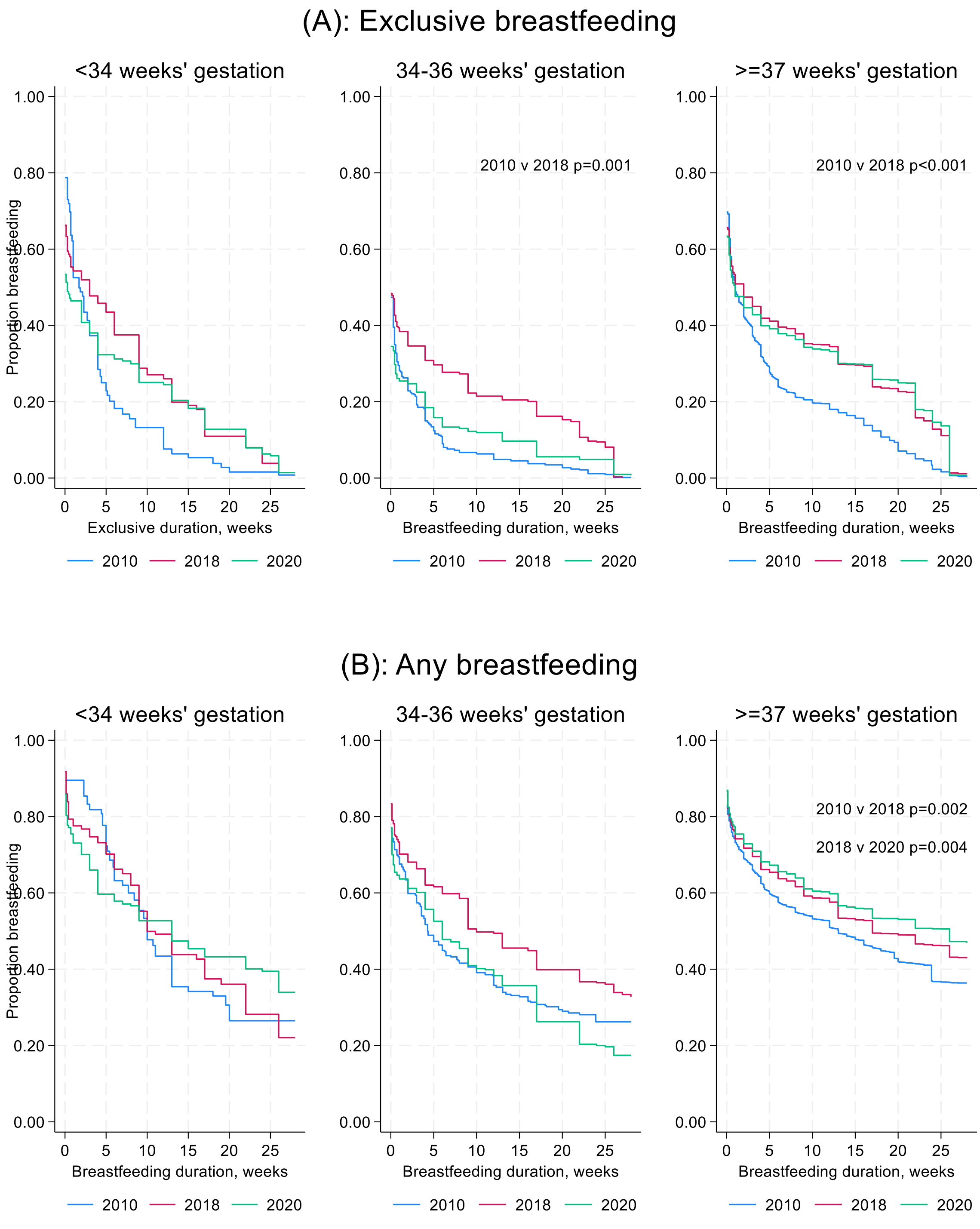

Results are weighted using each survey's individual weightings, and adjusted for maternal education, index of multiple deprivation, caesarean birth, parity and multiple birth.
