# Supplementary material for: Breastfeeding trajectories for preterm infants over the first 6 months of life in England 2010–2020: surveys using large representative birth samples
Source: BMJ Paediatr Open. 2024 Oct 21;8(1):e002912. doi: 10.1136/bmjpo-2024-002912 (PMC11499770; doi:10.1136/bmjpo-2024-002912)
Supplement: Supplementary file 2 [file bmjpo-2024-002912.reviewer_comments.pdf]

## PEER REVIEW HISTORY

BMJ Paediatrics Open publishes all reviews undertaken for accepted manuscripts. Reviewers are asked to complete a checklist review form and are provided with free text boxes to elaborate on their assessment. These free text comments are reproduced below.

## ARTICLE DETAILS

| | |
|---|---|
| **TITLE (PROVISIONAL)** | Breastfeeding trajectories for preterm infants over the first six months of life in England 2010-2020: surveys using large representative birth samples |
| **AUTHORS** | Levene, Ilana; Harrison, Sian; Alderdice, Fiona; Quigley, Maria A |

## VERSION 1 - REVIEW

| | |
|---|---|
| **REVIEWER NAME** | ***Kazuo Itabashi*** |
| **REVIEWER AFFILIATION** | None disclosed |
| **REVIEWER CONFLICT OF INTEREST** | |
| **DATE REVIEW RETURNED** | 02-Aug-2024 |

| | |
|---|---|
| **GENERAL COMMENTS** | Levine et al. identified challenges in breastfeeding support for LP infants on the basis that breastfeeding rates among LP infants showed an improving trend over time but, unlike term and very preterm infants, declined again in the SARs-Cov-2 pandemic. Furthermore, the paper is important because it suggests that LP infants may not be adequately supported with support similar to that provided to full-term infants.<br><br>The following points require amendment or comment.<br>(1) LP infants are those born between 34 weeks 0/7 and 36 weeks 0/7, and the more preterm of these infants may be affected by inadequate feeding support and have worse feeding outcomes. It would therefore be more reliable to show that there was no difference in the distribution of gestational weeks of LP infants included in the three study periods. The same applies to very preterm infants.<br>(2) In the devise, the authors state that the earlier discharge of LP infants compared to guidelines may have affected feeding outcomes, but if possible, they should indicate what the proportion of LP infants discharged earlier was in the three periods. |

| | |
|---|---|
| **REVIEWER NAME** | ***Karen M. Benzies*** |
| **REVIEWER AFFILIATION** | None disclosed |
| **REVIEWER CONFLICT OF INTEREST** | |
| **DATE REVIEW RETURNED** | 12-Aug-2024 |

| | |
|---|---|
| **GENERAL COMMENTS** | Dear Authors,<br>Thank you for the opportunity to review your manuscript reporting human milk feeding in preterm infants. In North America, it seems that researchers are now using the term "human milk feeding" rather |

| | |
|---|---|
| | than "breastfeeding" to be more gender neutral/sensitive. I do not have a preference, but thought I would bring to your attention.<br>In the abstract, suggest changing "change with the birth gestation..." to indicate lower gestation is associated with greater feeding challenges.<br>Response rates to the surveys were about 30%. I would be more convinced that the samples in the surveys were representative of the population if the authors presented a comparison of the socio-demographic characteristics of the samples and the general population at the times surveys were being conducted. It is unlikely that the samples included a representative proportion of preterm infants.<br>Overall, this is an important manuscript and highlights the need for greater feeding support for mother-late preterm infant dyads. |

**VERSION 1 – AUTHOR RESPONSE**

| | |
|---|---|
| Please give the ethics committee name and approval number in the main body of the text. | This information is available in the methods section on page 3 under the heading "Study Design". The approval number for the University of York ethics committee was not provided by the original authors so is not available. |
| LP infants are those born between 34 weeks 0/7 and 36 weeks 0/7, and the more preterm of these infants may be affected by inadequate feeding support and have worse feeding outcomes. It would therefore be more reliable to show that there was no difference in the distribution of gestational weeks of LP infants included in the three study periods. The same applies to very preterm infants. | This result is provided in Table 2 in the final two rows. The median and interquartile range for those born <34 weeks and 34-36 weeks is provided and the lack of a marker relating to statistical significance demonstrates that there was no significant difference in this distribution between years. Eg the median was 31 weeks and 36 weeks in all years for the two categories respectively. |
| In the devise, the authors state that the earlier discharge of LP infants compared to guidelines may have affected feeding outcomes, but if possible, they should indicate what the proportion of LP infants discharged earlier was in the three periods. | We have gone back to the original data to extract this information and added this data to table 2. We have amended the hypothesis accordingly, as there is no sign that mothers of LP infants specifically had earlier discharge in 2020, only term infants |
| In North America, it seems that researchers are now using the term "human milk feeding" rather than "breastfeeding" to be more gender neutral/sensitive. I do not have a preference, but thought I would bring to your attention. | We appreciate your comment and agree on the importance of inclusive language in this sphere. In this paper we have chosen breastfeeding due to word count restrictions. If the editors would like us to switch to gender neutral language, please advise if we can go over the word limits to do so. |
| In the abstract, suggest changing "change with the birth gestation..." to indicate lower gestation is associated with greater feeding challenges. | Unfortunately this is not a straightforward relationship. We describe in the introduction text that the mothers of the most preterm infants may have some advantages, in terms of motivation and staff support. This is reflected in |

| | |
|---|---|
| | the results, whereby late preterm infants had the worst outcomes of the three categories. With the word limits of the abstract we are not able to bring this complexity into this sentence. |
| Response rates to the surveys were about 30%. I would be more convinced that the samples in the surveys were representative of the population if the authors presented a comparison of the socio-demographic characteristics of the samples and the general population at the times surveys were being conducted. It is unlikely that the samples included a representative proportion of preterm infants. | Thank you, this is an important point. We have added text in the discussion highlighting the data available in previous publications and reports that tackles this question. We also have the following newly amended sentence in the discussion: “The proportion of late preterm infants in the sample is as expected with reference to Office for National Statistics data; 5% of births in 2010 were late preterm, 5.7% in 2018 and 5.3% in 2020; 2.2% of births in 2010 were at less than 34 weeks’ gestation, 2.2% in 2018 and 2.1% in 2020” – this can be compared by the reader with our results section which demonstrates that 4.9% of our sample were late preterm in 2010, 5.6% in 2018 and 4.9% in 2020; 1.2% of our sample were <34 weeks’ in 2010, 2.1% in 2018 and 2.7% in 2020. |
